# Annual Meeting of the American Medical Association

**Published:** 1868-06

**Authors:** 


### ANNUAL MEETING OF THE AMERICAN MEDICAL ASSOCIATION.

The Nineteenth Annual Session of the American Medical Association commenced Tuesday, May 5th, at 11 o'clock, at Carroll Hall, on G Street. The main portion of the hall is reserved for the use of the Association. The gallery is set apart for the use of the visitors, and was occupied during the session yesterday by a number of ladies and gentlemen.

The Convention was called to order at 11 o'clock, by the President, Dr. S. D. Gross, of Philadelphia, and prayer was offered by Rev. Dr. Pinckney, of this city, Washington, D.C.

The Permanent-Secretary, Dr. W. B. Atkinson, of Philadelphia, was at his post. Occupying seats on the platform, were Vice-Presidents Professor Post, of New York; Dr. Atlee, of Pennsylvania; Professor H. R. Storer, of Boston; and Dr. C. C. Cox, of Maryland.

The President next introduced Dr. Grafton Tyler, of Georgetown, who delivered the following address of welcome:

Mr. President and Gentlemen of the Association: The pleasant duty is assigned me to announce your welcome. After another year, spent in useful and laborious professional service, you are again assembled for all the pleasures and all the hopes of a social and a scientific reünion.

Your coming among us has been anticipated and provided for in the spirit of fraternity and of hospitality; but until this moment, when we see our wishes realized in this vast gathering of representatives from every part of our territory assembled before us, we knew not the fervor which inspires us now to greet you as we do, one and all, with the warmest welcome of our hearts. As I see before me representatives from the East, from the West, from the North, and from the South, I feel that the first pleasant promptings of duty overflow in a joyous enthusiasm. Welcome, then—thrice welcome among us; not as strangers, for the same enchanting power which bids you here binds us to a common brotherhood in science; not as foreigners, for this city founded by Washington, the father of his country, bearing his immortal name, is the home of all his country's people.

In greeting you, we also congratulate ourselves, not only on your presence here to-day, but upon the prospect of this being the place of your biennial meetings. What more appropriate selection than this centre of the national life—the seat of the National Government. You are not more the conservators of the life of an individual than you are of the life of a nation. Medicine, in its philosophy, its precepts, and its practice, is coëxtensive with the operations of government. It is exalted in the seat of justice, deciding, with inflexible sternness, the fate of innocence or guilt. It is sought and embraced for its indispensable aid, not only to the internal welfare, but to the national defence by sea and by land, as well as to its commercial wealth and its progressive civilization. The purposes of our profession are not less important than any which operate near the central power of a great nation, to give it perpetuity and exercise its efficiency. What more appropriate selection, then, than this as the depository of the archives, and the chief place for the assembling of this National Medical Congress. Just ten years ago, you honored our city with your presence, since which time much has been developed and improved of professional interest among us. Two medical schools vie with each other in honorable emulation, aided by several hospitals, which are advancing to stability in the promotion of the interests of science and of humanity.

While we can present to you but one institution as commanding in all respects as any of which the older cities can boast, perhaps inferior to none the world ever knew, to harmonize the broken, jarring links of intellect, you will have the rare opportunity to visit the depository of the accumulated gleanings of the battle-field and of the tented hospital—the worth of the noble sons of Hippocrates, whose temple you will see there adorned is more brilliant in the light of science, and as radiant in the glory of her achievements as ever Solomon's was with its gorgeous illumination. As you stand among those sacred relics in this hallowed temple, if associated reminiscences should touch your hearts, or scenes of strife in which you have mingled should rise up before you, the consciousness of having acted not to destroy, but to save—not to torture, but to soothe and comfort—not to wound, but to bind up, and staunch the flowing life blood—to cool the fevered lip—assist the suffering and solace the dying—will vindicate you as followers of the great physician "who went about doing good."

Does the martial hero rise to the pinnacle of this world's glory over thousands slain upon the battle-field? What greater glory his whose achievements, not only these, but in all his efforts and all his doings, secures alike to friend and foe only solace, comfort—aye, even life itself! The ministers of science, with earnest zeal and hopeful purpose, have made even a country's woes to contribute to the entertainment, the instruction, and good of all her people.

You are assembled here to-day to consider the most important interests confided to man, the health, the happiness, the social order, the domestic comfort of individuals, of communities, of nations, of the world—I say, of the world—for not only individuals, communities, and nations, but all mankind have received and acknowledged the blessing of American medical science. If we have received from abroad that great boon to man which has saved millions from deformity or death, we have given in turn that anæsthetic agent which, while it aids the efficiency of art, gives to all mankind freedom from pain, aye, not only is the surgeon's knife without this infliction, but the sorrow of woman, now tempered with joy, commands throughout the world a grateful homage to the genius of America.

In all the efforts of man, nothing has contributed so much to success as the principle of combination, and nothing has more secured the advancement of science than the establishment of associations and societies. The larger part of our knowledge is derived from comparing our own observations with those of others, and the mutual communication of our thoughts we solve many otherwise insuperable difficulties. I see now before me, the distinguished representatives of the universities, the hospitals, the societies, and associations of the cities of every State; I see before me, the representatives of the self-reliant, self-sacrificing, and no less honorable and efficient country practitioner; I see before me, the representatives of the army and navy, whose achievements in science cannot be eclipsed by the glory of war. Thus the profession of the whole country becomes associated for combined and harmonious effort in improving the result of their labors. For twenty-one years you have thus met together at great personal sacrifice—though sacrifice is common in your daily avocations, and attends all your paths to success. The history of medicine in this country affords nothing compared with these annual meetings, which has so much stimulated the industry, the generous professional and scientific ardor, the ethical spirit, and useful emulation of its votaries.

Well may the founders of this Association, who have been permitted to live so long, be this day proud of its record.

Thank God, that he who was "*primus inter pares*" is still spared to give us the joy of his genial presence and the blessing of his wise counsel and commanding influence, for whatever is now, or may be, recorded in the transactions of this Association, all that is profound in experimental research, all that is ingenious in discovery, all that is sublime in speculative science, all that is excellent for practical good, flowing to this common receptacle from the incitement of his forethought and the inspiration of his genius, while they distinguish their authors, become enduring monuments of his fame.

Under the guidance of well-disciplined minds, this Association, strictly national in its organization, and only professional in its aims and purposes, has singularly preserved its integrity, and, in confining its action to these purposes, has given an example worthy of imitation.

It is composed of the young and the old, of every positional grade—embracing always the great, the wise, and the illustrious—all of whom, as if inspired for the advancement and the dignity of the profession, though representing different States, with clashing opinions distinguishing them from one another, though divided in religious and political sentiment, and we may say even in views of social order, yet coming together as a common brotherhood in science, uniting in one grand concert to celebrate her truths, and in laying upon her altars the free gifts and never-fading offerings of wisdom and humanity.

While we feel the spirit of this sublime inspiration, we bid you again, one and all, the earnest welcome of our hearts.

At the conclusion of the address, which was warmly applauded, the President announced the programme, after which the Secretary called the roll, and most of the members responded. It was stated, however, that the entire delegation would be present to-day.

The next business in order was the presentation of members, and Dr. W. F. Percival, of Aiken, South Carolina, was admitted as a member by invitation.

A motion was made to postpone the delivery of the address of the President until to-day, but did not prevail, and Professor Gross then delivered his inaugural address, in which he occupied an hour. He returned his profound gratitude for the distinguished honor conferred upon him, in placing him in a position so often occupied by the distinguished men of the profession. For this mark of respect and confidence, he could only promise to do his duty impartially, and, as far as possible, expedite the business of the Association. The Professor then, at length, explained the object and ends of the Association, the duties of the profession, the advancement of the science, the brilliancy of the rank attained by the American faculty in the ranks of the profession throughout the world, the great good the Association had done in the past twenty years by its annual gatherings, and the continued good promised by its uninterrupted meetings. He gave, at length, his views on the reception of prize essays on medical subjects, and of the duties of professors of colleges, the management of hospitals, etc. He spoke in eloquent and impressive language of the departed members of the Association, and was exceedingly brilliant in his hopes and sanguine expectations of a bright future for this great Republic; entreated the great fraternity to go on doing good, as usual, throughout the length and breadth of the land, and concluded by again returning thanks to the Association for the honor they have conferred upon him.

At the conclusion of the address, on motion, it was ordered to be printed.

The regular business next followed—and the committees were called.

On Ophthalmology, Dr. Joseph S. Hildreth, Illinois, chairman; on Cultivation of the Cinchona Tree, Dr. J. M. Toner, District of Columbia, chairman; on Surgical Diseases of Women, Dr. Theophilus Parvin, Indiana, chairman; on Rank of Medical Men in the Navy, Dr. N. S. Davis, Illinois, chairman; on Insanity, Dr. C. A. Lee, New York, chairman; on American Medical Necrology, Dr. C. C. Cox, Maryland, chairman; on Leakage of Gas Pipes, Dr. J. C. Draper, New York, chairman; on Medical Ethics, ——— ———, chairman; on Plan of Organization, Dr. C. C. Cox, Maryland, chairman; on Provision for the Insane, Dr. C. A. Lee, New York, chairman; on the Climatology and Epidemics of Maine, Dr. J. C. Weston; of New Hampshire, Dr. P. A. Stackpole; of Vermont, Dr. Henry Janes; of Massachusetts, Dr. Alfred C. Garratt; of Rhode Island, Dr. C. W. Parsons; of Connecticut, Dr. E. K. Hunt; of New York, Dr. W. F. Thomas; of New Jersey, Dr. Ezra M. Hunt; of Pennsylvania, Dr. D. F. Condie; of Maryland, Dr. O. S. Mahon; Georgia, Dr. Juriah Harriss; Missouri, Dr. Geo. Engelman; Alabama, Dr. R. Miller; Texas, Dr. T. J. Heard; Illinois, Dr. R. C. Hamil; Indiana, Dr. J. F. Hibberd; District of Columbia, Dr. T. Antisell; Iowa, Dr. J. W. H. Baker; Michigan, Dr. Abm. Sager; Ohio, Dr. J. W. Russell; California, Dr. F. W. Hatch; Tennessee, Dr. Joseph Jones; West Virginia, Dr. E. A. Hildreth; Minnesota, Dr. Samuel Willey: on Clinical Thermometry in Diphtheria, Dr. Joseph G. Richardson, New York, chairman; on the Treatment of Diseases by Atomized Substances, Dr. A. G. Field, Iowa, chairman; on the Ligation of Arteries, Dr. Benjamin Howard, New York, chairman; on the Treatment of Club-foot without Tenotomy, Dr. L. A. Sayre, New York, chairman; on the Radical Cure of Hernia, Dr. G. C. Blackman, Ohio, chairman; on Operations for Harelip, Dr. Hammer, Missouri, chairman; on Errors of Diagnosis in Abdominal Tumors, Dr. G. C. E. Weber, Ohio, chairman; on Medical Education, Dr. A. B. Palmer, Michigan, chairman; on Medical Literature, Dr. Geo. Mendenhall, Ohio, chairman; on Prize Essays, Dr. Charles Woodward, Ohio, chairman.

Most of the committees responded as they were called, and their reports were referred to their appropriate sections.

The Committee on Medical Ethics, on Consultation with Female Practitioners, made a report, closing with the following resolutions:

*Resolved*, That the question of sex has never been considered by this Association in connection with consultations among medical practitioners, and that, in the opinion of this meeting, every member of this body has a perfect right to consult with any one who presents the "only presumptive evidence of professional abilities and acquirements" required by this Association, *viz.:* "a regular medical education."

*Resolved*, That the resignation of Dr. Julius Homberger, of New York, be accepted, and that all further consideration of him, or of his peculiar methods of procuring practice, be indefinitely postponed.

The report was laid over.

Drs. N. P. Tallifero and Buckner preferred charges against Dr. A. G. Field, of Iowa. The matter was laid over for investigation.

The Association then adjourned, to meet to-day at 9 o'clock.

In the afternoon, the several sections were engaged in listening to reports, and discussing the merits of the same.

The Committee on Prize Essays reported that four essays had been submitted, and the two prizes of $100 each were still in the hands of the Committee, as no awards had been made. They recommended that both should be offered for the best essay, but the subject was indefinitely postponed.

The general meetings of the Association will be restricted to the morning sessions, and the afternoon sessions, commencing at 3 o'clock, will be devoted to the hearing of reports and papers, and their consideration, in the following sections: 1. Chemistry and Materia Medica. 2. Practical Medicine and Obstetrics. 3. Surgery and Anatomy. 4. Meteorology, Medical Topography, and Epidemic Diseases. 5. Medical Jurisprudence, Hygiene, and Physiology. 6. Psychology.

The members of the Association called upon President Johnson last evening, and the East Room of the Executive Mansion was thronged until 9 o'clock with distinguished representatives of the medical profession from all parts of the country. Dr. Grafton Tyler presented the members to the President, who was assisted in the honors of the evening by Mrs. Stover and Mrs. Patterson, and Secretary Seward.

The reception at the residence of Hon. Schuyler Colfax took place from 9 to 10 o'clock, and after a cordial welcome on the part of Mr. Colfax, who was assisted by Mrs. and Miss Matthews, the company retired.

This evening the Association will call upon Senator Edwin S. Morgan, of New York. They will also spend several hours at the Army Medical Museum in examining the microscopical collections.

SECOND DAY.

Pursuant to adjournment, the Association met at 9 o'clock yesterday morning in Carroll Hall. There were present over three hundred delegates. Among the distinguished visitors who occupied seats on the platform, were Professors Stone, of New Orleans, Smith, of Baltimore, and Marsden, of Canada. Professor Gange, of the Prince Albert Veterinary College, London, the author of a number of valuable medical works, especially one on the rinderpest, was also an invited guest; and Senator Drake, of Missouri.

The report of the committee on the topics embraced in the President's address, was received, and the suggestions of the committee were ordered to be placed in the form of resolutions.

Dr. Cox, chairman on Alterations in the Constitution of the Association, and to revise the plan of organization, presented a report which advises many changes in the laws and orders governing the admission of members, and many amendments to the constitution.

The report was ordered to be printed, placed in the minutes of the proceedings, to be acted on at the next meeting of the Association.

A communication was received from the medical fraternity of New Orleans, inviting the Association to meet in that city at their next annual meeting. The communication was temporarily laid on the table.

A number of papers were received and referred to the various committees.

The Association then took a recess of fifteen minutes, so as to give the various State delegations an opportunity to select a member from their respective States to form the Nominating Committee for the ensuing year. This committee (one from each State) are empowered to nominate all officers for the Association.

At 10½ o'clock, the Association reässembled, when the various State delegations sent in the names of the delegates they had selected to form the Nominating Committee.

The following are the names selected: Maine, Dr. N. P. Monroe; New Hampshire, G. B. Switchell; Vermont, none; Massachusetts, H. R. Storer; Rhode Island, Bullock; Connecticut, Woodward; New York, Armsley; New Jersey, Lilly; Pennsylvania, Pollock; Delaware, Askew; Maryland, Hellsby; Virginia, Owen; West Virginia, Cummins; Georgia, Arnold; Ohio, Mussey; Illinois, Hildreth; Tennessee, John Keller; Alabama, Wetherby; Indiana, Sutton; Iowa, Cleaver; Michigan, Palmer; District of Columbia, F. Howard; United States Army surgeons, Otis.

After the reading of the names and their acceptance, it was resolved that the committee retire at once to organize.

While the committee were absent, several papers were received and referred to the various committees.

A number of names of physicians were received as candidates for membership to the Association. They were referred to the proper committee.

A letter was received and read, inviting the Convention to hold its next annual session at Fauquier White Sulphur Springs, Virginia. Referred.

Dr. Palmer, chairman of Committee on Medical Education, submitted a report of some length, which was listened to with marked attention by the Convention. It was referred to the Committee on Publication, and ordered to be printed.

On motion, Dr. Thomas J. Bunn, of the Choctaw Nation, was admitted as a delegate.

Dr. Mendenhall then submitted a written report, in detail, on medical literature. Referred to the Committee on Publication.

On motion, the communications in relation to the place of holding the next annual convention were taken up, and referred to the Committee on Nominations.

On motion, the Chair was authorized to appoint a committee of delegates to attend the Medical Convention at Montreal, Canada, in September next.

The report of the Committee on Medical Ethics, submitted Tuesday, was taken up and discussed.

The resolution was as follows:

*Resolved*, That the question of sex has never been considered by this Association in connection with consultations among medical practitioners, and that, in the opinion of this meeting, every member of this body has a perfect right to consult with any one who presents the "only presumptive evidence of professional abilities and requirements required by this Association," *viz.:* a regular medical education.

In support of the resolution, Dr. Washington Atlee, of Pennsylvania, delivered a vigorous speech, in which he advocated the recognition of female medical practitioners by the Association.

He spoke of the difficulties which had been thrown in the way of women making progress in their studies; but now they had a college of their own, which was free from all reasonable objections. In other countries, women had achieved the highest honors as medical practitioners, and he thought what could be done in France and Germany could most certainly be honorably done in the United States. He hoped the resolution would be adopted.

Dr. D. F. Condie, of Pennsylvania, next took the stand. It was his firm conviction that the mass of women would achieve higher honors in following the line of duty which had been marked out for them in the order of nature, and would do more good than in turning out physicians.

This remark was greeted with enthusiastic applause, but the Doctor begged them to keep their demonstrations of applause until he got through.

He proceeded to say: I deny that it is impossible for women not to be excellent physicians. There have been many eminent professional female medical practitioners in Europe, and the opportunities were equally favorable for the development of skilful and accomplished female medical practitioners in this country. But he thought this was a subject foreign to the interests of the American Medical Association. The question whether a male physician would consult with them ought to be left to individual judgment; let every member decide for himself. He then spoke of the situation in which one might be placed in an extreme case, where it was important, nay, absolutely necessary, that prompt action should be taken; but should the Association by its action condemn their recognition, what could a member of the Association do? He hoped the resolution would be laid on the table, and the course to be followed left entirely to individual discretion. The more it was discussed, and the more the Society opposed them, the greater would be the sympathy created for this class of the profession.

Dr. N. S. Davis, of Chicago, was the next speaker. His views of the subject were clearly defined, and he believed this Association had never taken action upon any matter which distinguished practitioners, either on account of sex or color. There was nothing in the laws of the Association forbidding any member from consulting with any qualified person one way or the other. It was of no importance who the party consulted was, provided they were duly qualified. That had become the universal standard.

If any local association saw fit to enact a law restricting its members, that was a matter for such societies to determine; but those so interfered with should not claim the legislative power of this Association to pass *ex post facto* laws for their especial benefit.

Dr. Davis then spoke of the dignity of woman, and the important part which she had at all times taken in the affairs of nations, and in all ages of the world. His respect, his reverence, his love, would forbid him drawing any distinctions between the sexes. Wherever and whenever humanity had needed her assistance, she was foremost in good works. The law of the Creator had assigned her sphere of duties; but if there was one who conscientiously believed that she could be of greater service as a physician, why should she be opposed. He was in favor of the broadest equality. If she was to be equal in the profession, let her be equal on the farm and in the ditch. Let the members consult with whom they please, if they are professionally qualified. He moved that the whole matter be indefinitely postponed.

The previous question being called for, the motion prevailed, with but few dissenting voices.

Dr. Atlee was anxious to say something, but the Chair ruled all remarks out of order.

Then, said Dr. Atlee, the Pennsylvania delegation are tied hand and foot.

The next business was the discussion of the following resolution:

*Resolved*, That the resignation of Dr. Julius Homberger, of New York, be accepted, and that all further consideration of him or of his peculiar methods of procuring practice be indefinitely postponed.

Dr. L. A. Sayre moved that the name of Dr. Julius Homberger be stricken from the rolls. He gave as his reason for such a motion, that the individual referred to had violated the code of ethics by which the members are bound to be governed. He also read a letter of inquiry from the editor of the *New Orleans Medical Journal*, concerning the standing of Dr. H., who was now in that city, advertising his "wares *in extenso.*"

Dr. Howard objected to the striking of Dr. Homberger's name from the roll on the testimony of a single accusation. He moved that this matter be referred to a committee of three, with instructions to investigate and report.

Dr. Arnold, of Georgia, denied that it was a single accusation. He stated that the testimony was abundant and well proven. The acts of this Dr. Homberger were disgraceful to the profession.

Dr. Noell, of Baltimore, obtained the floor and read the advertisement of several doctors and testimonials from physicians in practice of the qualities of certain quack medicines, and held that it would not be honest or proper to strike Dr. Homberger's name from the rolls until the skirts of the Association were clear of the same sin. It was a question of advertising, and he wanted it to be decided whether members of this Association should be permitted to lend their names to endorse the specialties of quack doctors.

Dr. Raphel, of Baltimore, thought too much importance was given to this question. Dr. H. had resigned, and it ought to have been accepted, though he was no doubt glad of the advertisement given him.

Dr. Davis, of Chicago, reviewed Dr. H.'s relations to the Society, and said the real question was, Shall a member who defied its rules be permitted to resign? This was last year referred to the Committee on Ethics, who reported the resolution. He thought the simplest plan to get rid of him and his humbugs was to accept his resignation.

Dr. Palmer, of Michigan, insisted that a resignation required the action of the Society. Dr. H. had violated its plainest rules. He should, therefore, be expelled.

The vote for the expulsion of Dr. H. passed without dissent.

Dr. Howard rose to make a personal explanation, to set himself right on the records of the Association. He did not wish to be understood as defending the course of Dr. H., but was opposed to hasty action, and wished also to learn the sense of the Association on the subject of advertising.

Dr. Hartman, of Baltimore, Maryland, submitted the following resolution:

*Whereas*, the third paragraph of the first article of the Code of Medical Ethics of the American Medical Association expressly declares it to be "derogatory to the dignity of the profession to resort to public advertisements, inviting the attention of individuals affected with particular diseases;" and,

*Whereas*, certain members of the medical profession in the city of Baltimore, who are permanent members of the American Medical Association, have permitted their names to appear in the daily newspapers endorsing the qualifications and professional character of a foreign specialist who has recently settled here ; therefore,

*Resolved*, That by such conduct these gentlemen have been accessory to a violation of professional ethics, and guilty of an unwarranted and unjustifiable discrimination against those members of the profession who are quietly, legitimately, and unostentatiously prosecuting the respective branches of medical specialism.

*Resolved*, That is the sense of the Baltimore Medical Association that either of the above paragraphs of the code of ethics should be so modified as to allow our own professional brethren who are engaged in the practice of specialties to advertise, and thus be placed on an equal footing with the foreign specialist, who is too often a mere adviser, whom chance has thrown among us; or those gentlemen who have permitted the use of their names to swell the patronage of a special specialist, to the injury of equally deserving members of the profession in our own city, should be compelled to withdraw their names from such advertisements.

*Resolved*, That the delegates to the American Medical Association from this Society be instructed to bring this matter, so vital to the dignity and honor of the profession, before that body at the earliest possible moment.

The paper was referred to Committee on Ethics, after which the Association adjourned until to-day, at 9 o'clock.

The microscopical exhibition in the lower hall of the Army Medical Museum, on Tenth Street, last evening, was one of the finest ever witnessed in the United States. The entire delegation was present, as well as a large number of the prominent Government officials. Previous to the exhibition, the guests spent several hours in examining the large collection of anatomical specimens collected in the upper halls.

The exhibition was conducted by Dr. J. J. Woodward, and proved most interesting to all present. The members of the medical faculty manifested their admiration at the success attained in photographing anatomical specimens by their enthusiastic applause.

The enjoyments of the day terminated with a brilliant reception at the residence of Senator Morgan.

This evening the Association will be received by Chief-Justice Chase and Mayor Wallach.

THIRD DAY.

The Association resumed its session yesterday morning at nine o'clock, the President, Dr. S. D. Gross, in the chair. The attendance of delegates was about the same as on the preceding days. There were a few visitors in the gallery. A number of valuable works on medical subjects were gratuitously distributed among the members of the Association, as were also samples of new and improved medicinal preparations.

A letter was read from Dr. Cornelius Boyle, of Fauquier, White Sulphur Springs, Virginia, inviting the Association to hold its next annual session at that place; which was referred to the committee on nominations.

The reports of the treasurer and publication committee were then read and accepted.

The report of the committee on nominations being in order, the same was presented, and after some debate it was accepted. The report names New Orleans, Louisiana, as the place to hold the next meeting of the Association, and fixes the time for May next. The following officers of the Convention were nominated by the committee:—President, Dr. Wm. O. Baldwin, of Alabama; 1st Vice-president, George Mendenhall, of Ohio; 2d

Vice-president, Noble Young, of Washington, D.C.; 3d Vice-president, Dr. N. P. Monroe, of Maine; 4th Vice-president, Dr. S. M. Bemis, of Louisiana; Treasurer, Dr. Caspar Wistar, of Philadelphia, Pa.; Committee on publication, Dr. Francis G. Smith, Jr., of Philadelphia, (chairman); Dr. Wm. B. Atkinson, of Philadelphia; Dr. H. F. Askew, of Delaware; Dr. Richard M. Cooper, of New Jersey; J. H. Lovejoy, of D.C.; Dr. Wm. Mabury, of Pennsylvania.

Dr. Mabury offered an additional amendment to article five, plan of organization, "No report purporting to emanate from any committee shall be received, unless it be signed by a majority of its members." Laid over.

The secretary suggested to the delegates that the business of the publication committee was rapidly on the increase, and that the funds on hand were not adequate to meet the expenses of printing all the proceedings as they should be.

The committee on the President's address made their report, accompanied by the following resolutions:—

*1. Resolved,* That the publishing committee are hereby invested with plenary power in regard to all papers not read before the Association or in the section, to publish or not, as may seem expedient.

*2. Resolved,* That a committee of three be appointed by the Chair, to take into consideration the subject of appointment of a commissioner in each judicial district or circuit, whose duty it shall be to aid in the examination of witnesses in every trial involving medico legal testimony, and to report at the next meeting of the Association.

*3. Resolved,* That a committee be appointed to report next year, in regard to the subject of an annual register of the regular profession in the United States, and in the meantime to take necessary measures to carry the plan into effect.

*4. Resolved,* That a committee be appointed to take into consideration the subject of the best mode of providing a fund for the relief of widows and orphans of deceased physicians, and report to the Association at the next meeting.

*5. Resolved,* That a committee of three be appointed to take into consideration the subject of the establishment of veterinary colleges, and report at our next meeting.

*6. Resolved,* That all hospitals and public institutions for the care and treatment of the sick should have educated, well-trained nurses only; that this Association would strongly recommend the establishment in all our large cities of nurse training institutions.

The first five resolutions were adopted, and the sixth was referred to a special commitee, consisting of Drs. S. D. Gross, of Philadelphia; Elisha Harris, of New York; and Charles Lee, of New York.

The Chair then announced the following committees:—

Commissioners to Aid in Trials Involving Scientific Testimony—Drs. John Ordeonaux, of New York; A. B. Palmer, of Michigan; Stephen Smith, of New York; J. W. Dunbar, of Baltimore.

Annual Medical Register—Drs. Packard, of Philadelphia; Wm. B. Bibbins, of New York; and Ellsworth Eliot, of New York.

Devising a Plan for the Relief of Widows and Orphans of Medical Men—Drs. J. H. Griscom, of New York; N. S. Davis, of Illinois; and A. C. Post, of New York.

Veterinary College—Drs. Thos. Antisel, of Washington, D. C.; C. C. Lee, of New York; and John C. Dalton, of New York.

He also appointed the following delegates to represent the American Medical Association in Canada, to meet in September next—C. C. Cox, M.D., LL.D., of Maryland; Drs. John L. Atlee, of Pennsylvania; N. S. Davis, of Illinois; Charles A. Lee, of New York; Grafton Tyler, of D.C.; W. M. Wood, of the Navy; and S. D. Gross.

On motion of Dr. Howard, of Maryland, the following gentlemen were appointed to prepare and submit at the next meeting of the Convention, a report on the subject of specialties in medicines—Drs. E. Lloyd Howard, Frank Donnelson, and Christopher Johnson, all of Maryland.

Dr. C. C. Cox, of Maryland, then read the report on American medical necrology, which occupied some time, and was ordered to be printed.

There were several resolutions offered, and appropriately referred.

At 12 o'clock, Dr. Atlee, of Pennsylvania, escorted to the platform the newly elected President, Dr. William O. Baldwin, of Montgomery, Alabama.

The appearance of these gentlemen was the signal for enthusiastic applause, and when silence had been restored, Dr. Atlee introduced Dr. Baldwin to the Convention through the retiring President, Dr. Gross, and the manner in which the latter welcomed his successor thrilled the hearts of all present with patriotic joy. Dr. Gross said:

I welcome you as the representative of our long lost brethren. May God bless you; God bless your people; God bless all of us.

Dr. Baldwin then delivered the following address:

Mr. President, and Gentlemen of the American Medical Association:—In returning you my sincere thanks for the honor you have conferred upon me in electing me to preside over the deliberations of this body—an Association which embraces in its membership so many names justly distinguished over the civilized world for genius and learning—believe me, gentlemen, it is with feelings of embarrassment equalled only by my profound sense of gratitude and my admiration for the magnanimity which prompted the offering. It is the more grateful to me that it was the free, unasked-for gift of the Association. I did not seek the position. High as the honor is, I should deem it purchased at too dear a price, if, in order to obtain it, it had been necessary for me to solicit the votes of any men from any section, even those from my own society.

I am painfully conscious, gentlemen, of my own unworthiness of the high distinction, and am not vain enough to appropriate the honor all to myself. I do not accept it as an individual compliment, but rather as the faithful hand of brotherhood stretched out with a generous friendship and true nobility of soul in its desire to heal and obliterate the wounds in its own bosom, for whose creation it was in no way responsible.

Pardon me for taking this opportunity for alluding briefly to a subject which has not, perhaps, heretofore been considered germane to occasions like the present, and which I now approach with both pain and hesitation. I am sure that most of you have not failed to observe the very meagre representation which this Association has had from the Southern States since the close of the late war. This has probably been due to several causes, to only one of which, however, I desire to allude. I will not disguise from you, gentlemen, the fact that there are many physicians in the South disposed to hold themselves aloof from your councils.

The resolution passed at your meeting in 1866, offering again the hand of fellowship for your Southern brethren, owing to the peculiar condition of our country at that time, and the fact that but little of the medical literature and news of the North met the eyes but of few, and there are still among us those who feel that your hearts are still stolid against them, and who believe that, notwithstanding some formal declarations to the contrary, most of you, in your private feelings, have not yet been able to rise sufficiently above the prejudices of the past to enable you to receive them in such a manner as to make their presence here either agreeable to them or profitable to the Association.

Looking to this conviction of theirs, strengthened by the fact that they are still under the cloud of the nation's displeasure, and denied the political rights to which they esteem themselves entitled, they have felt that it would be both undignified and unmanly to present themselves at your doors for admittance to your councils, or to offer to affiliate with you, until they can come as your peers in all things—in political and social rights, as well as in scientific zeal and devotion.

So far as my observation has extended, I am sorry to know these sentiments have prevailed with many, and it is but frankness in me to say so. I am free to confess that I, with many others, have not sympathized altogether with these feelings. I saw the resolution adopted in 1866, and before referred to, inviting us, in most respectful and conciliatory language, to resume our places in this Association. I felt this was all you could do, all you ought to do, all we could ask, and was satisfied with it, and only regret it did not obtain a more general circulation. The society to which I belong, with entire unanimity, appointed its full quota of delegates to this meeting. I came here to lend my humble example to the work of reëstablishing our former relations. I never doubted I would be received with courtesy, and even with kindness.

The broad, liberal, and catholic sentiments proclaimed from this stand, in the annual address of that noble old Roman, our distinguished President, Dr. Gross, knowing in these halls "no North, no South, no East, no West"—he whose clustering honors, though won in your midst, yet gather a beauty and brilliancy from the love and veneration in which he is held in the South—must be received as a declaration of sentiments and principles by this Association, and cannot fail to correct the errors and misrepresentations which have prevailed in our section. This action of yours to-day, in awarding, through me as one of her humble representatives, the honorable and distinguished office of president of this Association, a position which might well be claimed for one of the many of your own renowned and gifted sons, will, I am sure, testify to our brethren of the South, in silent but forcible language, the injustice which has been done you by those who have taken a different view of your real sentiments and feelings towards us.

In saying this much I do not intend it as a reproach to those of my section who have hitherto so misunderstood you, and you, in your generosity, I am sure, are prepared to concede much to the pride of a noble manhood, who, standing amidst the memories of blasted hopes and ruined fortunes, have, perhaps, been disposed to guard with too jealous and sensitive an eye that which is dearer to them than fortune or life itself, and which I am sure you would be the last to willingly see compromised—their personal and professional dignity and honor.

For myself, and for those whom I represent, I grasp with unaffected pleasure the hand which you have so gracefully and magnanimously offered, and I hope and believe this sentiment will meet a ready response from all our brethren of the South. *Let us again be united as friends and brothers.* Ignoring past and present political difficulties, let us exhibit to this distracted country an example of forgiveness and toleration worthy the emulation of a great and noble people. Let the bonds which we acknowledge here bind us in all portions of this broad land as a sacred brotherhood, engaged in a common toil, with one mind, one heart, and one purpose. Let the place annually selected for our meetings be our Mecca. There let us meet with harmony of sentiment for thorough organization, for connected and concerted action, without which no great science or art can ever attain its highest perfection. Exacting from each other only the qualifications necessary for honorable membership, let us there mingle in the sacred precincts of our humane profession, and join hands and sympathies in the strengthening influences of association and fellowship; and as we lay fresh offerings in the temple of a noble science, and build new fires on her altars, let us cherish in our hearts the ennobling sentiment of brotherly love.

In conclusion I would say we have doubtless most of us, aye, certainly most of us, in that land of many sorrows from whence I came, tasted the bitter fruits of the bloody and unholy war through which we have passed, and wept over its dire calamities. *We*, as an association, had no agency in its creation. It belongs now, with all its disasters and miseries, to the dead past, and as *we* had no cause for quarrel then, we have none now for separation or estrangement. We may not forget our sorrows for the past, and we will still water with our most sacred tears the graves of our noble sons who fell victims to the strife. But whenever there is a grief at the heart, a tear for the ashes of the past, let us wipe from it all traces of bitterness, and drape its memories and sanctify its sadness with the Christian virtues of CHARITY, FORGIVENESS, and FRATERNAL LOVE.

During its delivery the speaker was frequently interrupted with applause, and on concluding was the recipient of the hearty congratulations of those on the platform.

The President, Dr. Gross, said he desired to avail himself of this opportunity to correct an erroneous statement which had gained publicity throughout the Southern States, in regard to a resolution alleged to have been passed by this Association, recommending that the Government should make surgical instruments and medicines contraband of war. He said, I take this occasion to deny that the American Medical Association ever passed any such resolution, and hope our President elect will do every thing in his power to promulgate this fact among our Southern brethren.

Dr. Davis desired to say in addition, that not only had no such resolution ever been adopted, but that it had never been introduced.

This statement was, on motion, ordered to be recorded in the the transactions of the Association.

An invitation was read from the Young Men's Christian Association, of this city, for the Medical Association to visit their library and reading-room.

On motion, the committee on archives was continued.

On motion, the secretary was instructed to appoint a sub-committee of arrangements of three from each State.

Dr. N. S. Davis, of Illinois, offered a resolution instructing the Chair to appoint a committee of three, to report at the next session, on the practicability of establishing a library of American medical works, including books, monographs, and periodicals. Adopted.

The Association then adjourned till nine o'clock to-morrow.

In accordance with the invitation extended to the members of the Association, they assembled at the Capitol last evening, to witness the illumination of the dome. The several sections were lighted, and the manner in which the electrical apparatus is worked was explained by Prof. Gardner, to the ladies and gentlemen present.

The reception at the residence of Chief Justice Chase was attended by the delegates in a body, and an hour was pleasantly passed in conversation and in enjoying the hospitality of the house.

The reception at the residence of Mayor Wallach took place at 10 o'clock, and was attended by all the delegates. It formed a brilliant conclusion to the cordial welcome extended to the distinguished representatives of the profession by the people of Washington. The remainder of the evening was spent in social enjoyment. A sumptuous repast was provided, and the party did not separate until a late hour.

FOURTH DAY.

The Convention resumed its session yesterday morning at 9 o'clock, Dr. Gross in the chair.

Dr. Tyler read an invitation from Mr. Sykes, inviting the Convention to visit Mount Vernon, at half-past 11 o'clock, on his steamer; which was accepted.

He also read an invitation from Mr. King, requesting that he be allowed to make a photograph of the Convention; which was accepted, and at 10 o'clock the picture was made in front of the hall.

Dr. Martin, of Massachusetts, offered the following, which was adopted:

It seems proper that this Association should not be without a committee on a subject so transcendantly important as that of vaccination; therefore

*Resolved*, That a standing committee of one be appointed upon the whole subject, to report from time to time on such topics connected with vaccination as shall, in the estimation of such committee, appear of chief practical interest and importance to the profession.

Dr. Antisell, of this city, was appointed as the committee.

The committee on nomination made the following report, which was adopted:

Assistant Secretary—Dr. A. G. Semmes.

Committee of Arrangements—Drs. J. G. Richardson, S. M. Bemis, C. Beard, L. T. Pimm, Warren Brickell, S. Chopin, and —— Mitchell.

On Medical Education—Drs. J. C. Reeve, Dayton, Ohio; J. S. Hildreth, Chicago; W. D. McCook, Pittsburg, Pa.; Frank Rice, Memphis, Tenn.; and S. H. Pennington, Newark, N.J.

Committee on Necrology—Drs. S. S. Cox, Maryland; E. B. Stevens, Ohio; W. F. Peck, Iowa; H. Van Dozen, Wisconsin; J. M. Toner, District of Columbia; Joseph Simpson, United States Army; J. C. Weston, Maine; Henry Bronson, Connecticut; Henry Noble, Illinois; Charles Eversfield, United States Navy; T. Parvin, Indiana; J. C. Hupp, West Virginia; Joseph Mauran, Rhode Island; J. M. Keller, Tennessee; H. T. Asken, Vermont; H. J. Clark, Massachusetts; E. M. Moore, John Shrady, New York; Charles A. Logan, Kansas; —— Stewart, Minnesota; Henry Miller, Kentucky; F. G. Armour, Michigan; John Blaine, New Jersey; A. Fleming; Howard Wallace, Pennsylvania; R. D. Arnold, Georgia; J. S. Wetherly, Alabama; S. L. Welch, Texas; T. M. Logan, California; John W. H.

Baker, Iowa; P. A. Stackpole, New Hampshire; L. Joynes, Virginia; W. Brickell, Louisiana; David Booth, Mississippi.

Committee on Literature—Drs. Edward Warren Baltimore; Joseph Jones, Nashville; Edmund Andrews, Chicago; J. J. Woodward, United States Army; P. S. Wales, United States Navy.

Committee on Climatology—Drs. J. C. Weston, Maine; P. A. Stackpole, New Hampshire; Henry James, Vermont; Alfred C. Garrett, Massachusetts; C. W. Parsons, Rhode Island; E. K. Hunt, Connecticut; W. T. Thomas, New York; E. Hunt, New York; D. T. Condre, Pennsylvania; O. S. Mahon, Maryland; J. Harris, Georgia; George Engleman, Missouri; R. F. Michel, Alabama; T. J. Heard, Texas; R. C. Hamill, Illinois; J. F. Hibbard, Indiana; T. Antisell, District of Columbia; J. C. Hughes, Iowa; Abraham Sagur, Michigan; T. L. Neal, Ohio; F. W. Hatch, California; B. W. Avent, Tennessee; E. A. Hildrath, West Virginia; —— Owen, Virginia; Samuel Willey, Minnesota; L. B. Bush, Delaware; G. W. Lawrence, Arkansas; —— Comptin, Mississippi; Louis Pimm, Louisiana.

Committee on Prize Essays—S. M. Bemis, J. Scott, W. Brickell, S. A. Smith, C. Beard.

Special Committee on Alcohol and its relations to Medicine —John Bell, J. R. Dunbar, and Richard McSherry.

On Cryptogamic Origin of Disease, with special reference to recent microscopic investigations on that subject—Dr. Curtis, United States Army.

On Diseases of the Cornea—Dr. J. S. Hildreth, of Chicago.

On Excision of Joints for Injuries—Dr. J. B. Reed, of Savannah.

They also reported the following resolution, which was adopted:

*Resolved,* That those gentlemen who desire to report on special subjects, and will pledge themselves to report at the next meeting, be requested to send their names and the subjects they desire to report upon to the Secretary of the Association.

Dr. C. A. Lee, of New York, was appointed a delegate from the Convention to the meeting of superintendents of insane asylums.

Professor Henry, of the Smithsonian Institute, was invited to a seat on the platform.

The paper submitted by Dr. Joseph Jones on Albinism in the negro race, was recommended for publication from the Smithsonian Institute.

The thanks of the Convention were returned to the Baltimore and Ohio railroad; Philadelphia, Wilmington, and Baltimore road; Orange and Alexandria, and other railroads, for facilities shown to the members of the Convention.

The thanks of the Convention were also tendered to the President of the United States, Speaker Colfax, Chief Justice Chase, Hon. Richard Wallach, Professor Samuel Gardiner, electrician United States Capitol; Dr. Woodward, of the Army Medical Museum; the committee of arrangements, of which Dr. Tyler is Chairman; and Hon. Edwin D. Morgan, for their politeness and courtesies to the Association. Also to the press of the city for the correct reports of the proceedings of the Convention.

The President appointed the following gentlemen as delegates to foreign medical societies: Samuel J. Jones, of Chicago; G. C. Blackman, of Cincinnati; Fordyce Baker, of New York; to which committee Dr. Gross, President of the Convention, was added.

The President read a letter from Hon. George Bancroft, our Minister at Berlin, relative to Professor Aaron Burke, the great microscopist, who is now blind.

On motion, Dr. Gross, the President, was authorized to send a letter on behalf of the American Medical Association, complimentary of Professor Burke.

Drs. Howard and Dunbar, of Baltimore, and C. A. Nichols, of this District, were appointed as a committee on the subject of marine hospitals.

Dr. Wetherly, of Alabama, spoke of the next meeting of the Convention, and declared that he had no doubt the members would be gladly welcomed and as hospitably entertained in New Orleans as at any place where they had ever met. [Applause.]

Dr. Gross then arose and spoke as follows: Before the question of final adjournment be put, allow me to tender you my cordial acknowledgments for the kindness and courtesy which you have extended to me as your presiding officer. Gratitude and good taste alike prompt the expression of my feelings. In everything I did I felt that I had your generous support and sympathy; whatever errors may have been committed were errors of the head and not of the heart, and are, I am sure, already forgotten by you. I congratulate you upon the manner in which you have conducted your proceedings. It is questionable whether there ever was a deliberative body of such magnitude in which there was so little discord, or so little said and done of an objectionable character. Harmony, cordial and complete, prevailed from the beginning to the end. There was, indeed, not one word uttered that any one, even the most fastidious, might wish to recall; a circumstance the more surprising when it is recollected that men in the heat of debate often give way to heedless and unguarded expressions calculated to ruffle the feelings, and to engender unpleasant reminiscences. We have accomplished not a little work, and, above all, we have had an opportunity of reviving friendly feeling, of extending our acquaintance with each other, and of interchanging sentiments in regard to matters of vital importance to our beloved profession. I am sure that every one will say, as he leaves this hall, that it was good for him to have been here, and that he will return to his home with new resolves, and determined to devote himself more earnestly than ever to the advancement of the glory of his noble calling; that he will strive more than ever to elucidate its great principles, and that, abandoning all other pursuits, he will worship medicine as the only goddess of his idolatry. Hoping that no evil may befall you on your homeward journey, and that your families may greet you with messages of peace and glad tidings, I bid you a cordial and affectionate farewell.

The Convention then adjourned *sine die.*

### THE EXCURSION TO MOUNT VERNON.

About noon yesterday, the fine steamer Arrow, having on board a large number of the delegates to the Convention, with a large party of ladies, left her wharf for an excursion trip to Mount Vernon.

The weather was delightful, the sun shone brightly, and from the arrangements which had been made, a very pleasant trip was anticipated. Professors Frank Malone and J. P. Spannier, with several members of Heald's Brass Band, were on board, and on the way down performed many choice selections. At Fort Washington, the fine brass band of that station, recently organized under the tutorship of Professor Blaw, embarked, and aided with their sweet music in adding to the pleasures of the day.

The boat with the excursion party arrived at Mount Vernon about 2 P.M., and at once all betook themselves to the task of seeing all that there was to be seen. The first place visited was, of course, the vault in which lie the remains of the Father of his Country, and his wife, Martha Washington. The mansion and grounds, and every point of interest in and around the place was glanced at hurriedly, of course, as but one hour was allowed, in order that the delegates could reach this city in time to leave by the evening trains. At 3 o'clock, the party embarked, and after the steamer was under way the delegates were called together on the upper deck, when Dr. L. A. Sayre, of New York, addressed the meeting:

In leaving the hallowed spot where rests all that is mortal of the greatest and best of men—the tomb of Washington—is it not eminently befitting such an occasion that we should renew our vows of patriotic devotion at the shrine of him who is justly called "The Father of his Country?" The fervent prayer inspired in every loyal heart, while contemplating the scene before us, must be that the spirit which guided him through life may be kindled in each one of us. The principles of patriotic love of country, of unbounded fealty to the Union, which characterized the life of Washington, are bequeathed to us as the inheritance of every true American citizen.

The American Medical Association, organized for the purpose of promoting the best interests of the profession throughout the land, is governed by principles looking only to the good of humanity, and cannot possibly entertain sectional distinctions or geographical lines. We recognize all as our brethern. The example which as a body has been given, in choosing as our next place of meeting the city of New Orleans, is in itself worthy of imitation by those who profess, as national legislators, to have only the good of the nation at heart.

But to this we have added another evidence of the sincerity of our good will, for in conferring upon a citizen of the South the highest position the Association can offer, we have positively asserted our faith in the people of the South to stand by the Union, and to remain unwavering in their fidelity to the laws of the land. Let this be at least in some measure a lesson to those who are endeavoring to heal the wounds of the nation by political legislation.

Dr. N. S. Davis, of Chicago, the founder of the American Medical Association, was then called upon, and responded in an eloquent and patriotic address. He was followed by Dr. W. O. Baldwin, of Montgomery, Alabama, the President elect of the Association, who said that the medical profession of the South would henceforth be a unit in the work of the Association.

Appropriate addresses were also delivered by Drs. Hibbard, of Indiana; Hooper, of Massachusetts; Condie, of Philadelphia, and Harris, of New York city.

The recently invented steam guage of Mr. D. M. Greene, which has been placed in use on board of the steamer Arrow, was examined by the many scientific gentlemen present with a great deal of interest.

Shortly before the boat reached the wharf the following card of thanks was prepared, and after being signed by the distinguished gentlemen whose names are annexed, was presented to Mr. Sykes. The following is the card:

"We, the undersigned delegates of the American Medical Association, tender to Mr. James Sykes and Captain Thomas Stackpole, of the steamer Arrow, our sincere thanks for the kindness and courtesy shown to us in our visit to the tomb of the father of our country.

"The beautiful application of the science of chemistry which these gentlemen have applied to their boat in the 'thermometric steam guage,' the invention of Mr. D. M. Greene, rendering accidents from the explosive power of steam impossible, has given us a sense of security which has added materially to our pleasures."

Signed by Dr. N. S. Davis, of Chicago; Dr. W. O. Baldwin, of Alabama; Drs. H. N. Eastman; T. M. Hooper, of Massachusetts; Foster Hooper, Joseph Taber Johnson, Lewis A. Sayre, of New York city; J. H. Buckner, Henry A. Martin, T. J. Thomason, H. V. M. Miller, Joseph Swartz, John H. Packard, J. C. Dorr, J. F. Jarvis, S. S. Bates, G. W. Barr, H. J. Norton, J. P. Root, J. W. Hopton, H. A. Robbins, C. H. Bowen, Augustus Mason, J. M. Lazzell, J. M. Toner, C. D. Norton, Francis C. Condie, W. H. Pancoast, R. R. McIlvaine, Wm. F. Tomes, James F. Hibbard, J. W. H. Lovejoy, and Dr. E. Harris, whose name is familiarly known to the scientific world in connection with investigations on the cause of epidemics.

About half-past five o'clock, the boat with its precious freight arrived at the wharf, and the party disembarked, all expressing their great gratification at the very pleasant time enjoyed by all who participated in the pleasures of the day.

Messrs. Sykes, the proprietor, and Stackpole, the commander of the steamer, were unremitting in their endeavors to please all of their patrons, and to their exertions are due much of the satisfaction experienced by the excursionists on their trip.

The most of the delegates left the city last evening for their homes. We understand that all of the gentlemen who attended the Convention have expressed themselves highly pleased with the attentions received while on their visit to this city.

